# Autosomal Dominant Retinitis Pigmentosa Secondary to TOPORS Mutations: A Report of a Novel Mutation and Clinical Findings

**DOI:** 10.3390/jcm13051498

**Published:** 2024-03-05

**Authors:** Alen T. Eid, Kevin Toni Eid, James Vernon Odom, David Hinkle, Monique Leys

**Affiliations:** 1 Department of Ophthalmology and Visual Sciences, West Virginia University School of Medicine, Morgantown, WV 26506, USA; jodom@hsc.wvu.edu; 2 Department of Ophthalmology and Visual Sciences, John A. Moran Eye Center, University of Utah, Salt Lake City, UT 84112, USA; keid@oakland.edu; 3 Tulane University School of Medicine, New Orleans, LA 70112, USA; davidhinklemd@gmail.com

**Keywords:** TOPORS, retinitis pigmentosa, OCT, retina, ADRP, Appalachia, inherited retinal disease, retinal degeneration, RP, genetics, mutation, macular hole

## Abstract

**Purpose:** Mutations in Topoisomerase I–binding RS protein (TOPORS) have been previously documented and have been described to result in pathological autosomal dominant retinitis pigmentosa (adRP). In our study, we describe the various genotypes and clinical/phenotypic manifestations of TOPORS-related mutations of our unique patient population in Rural Appalachia. **Methods:** The medical records of 416 patients with inherited retinal disease at the West Virginia University Eye Institute who had undergone genetic testing between the years of 2015–2022 were reviewed. Patients found to have pathologic RP and mutations related to TOPORS were then analyzed. **Results:** In total, 7 patients (ages 12–70) were identified amongst three unique families. All patients were female in our study. The average follow-up period was 7.7 years. A mother (70 yr) and daughter (51 yr) had a novel heterozygous nonsense point mutation in TOPORS c.2431C > T, p.Gln811X (Exon 3) that led to premature termination of the desired protein resulting in early onset vision loss, cataract formation, and visual field restriction. The mother developed a full-thickness macular hole which was successfully repaired. Five other patients were found to have previously described TOPORS mutations. Visual field loss was progressive with age in both cohorts. **Conclusions:** Seven patients at our institution were identified to have mutations in TOPORS resulting in autosomal dominant retinitis pigmentosa. Two patients were found to have novel truncating mutations in the TOPORS gene resulting in profound night blindness and visual field loss, recurrent macular edema, and in one individual, epiretinal membrane formation leading to a macular hole which was able to be successfully repaired.

## 1. Introduction

Retinitis pigmentosa (RP) is a group of inherited retinal dystrophies characterized by the progressive degeneration of photoreceptors and retinal pigment epithelium (RPE), leading to night blindness, visual field constriction, and eventual loss of central vision. RP affects approximately 1 in 4000 individuals worldwide and is genetically heterogeneous, with more than 80 genes implicated in its pathogenesis [1,2,3]. Autosomal dominant RP (adRP) accounts for about 15–30% of all RP cases and is caused by mutations in at least 25 genes [1,3,4].

One of the genes associated with adRP is TOPORS, which encodes topoisomerase I-binding RS protein, a dual E3 ubiquitin and SUMO1 nuclear protein ligase that is involved in cell cycle regulation, protein synthesis, DNA repair, and chromatin modification in different cell types [3,4,5,6]. The gene itself is present on the short arm of chromosome 9 band 21, is roughly 12,000 base pairs in length, and encodes a ubiquitous multidomain SUMO1-Protein E3 ligase protein comprised of 3 exons. Mutations in TOPORS have been reported to cause adRP linked to chromosome 9p21.1 (locus RP31) in several families from different ethnic backgrounds. Pathologic mutations in the retina eventually lead to loss of the outer portions of the photoreceptors which is likely related to the disruption of cilia-dependent photoreceptor cell maintenance pathways [4,7]. The majority of these mutations are located in the terminal half of exon 3 and result in premature truncation or missense changes of the TOPORS protein.

The phenotypic features of TOPORS-related RP may include early onset of night blindness, severe progressive visual field constriction, cystoid maculopathy (CM), and reduced electroretinogram (ERG) stimulatory responses. Given the rare nature of TOPORS-related RP, genotype–phenotype correlations of TOPORS mutations and the exact molecular mechanisms behind retinal degeneration caused by TOPORS dysfunction are still not fully understood [3].

In this case, we report the prevalence and characterization of TOPORS mutations in a cohort of patients with inherited retinal disease from rural Appalachia. We identified seven patients from three separate families with mutations in TOPORS, including the identification of a novel nonsense mutation that causes premature truncation of the protein. Their clinical course and examination findings are described in detail below. Lastly, we describe an isolated case of full thickness macular hole that was successfully repaired and was able to restore limited vision in one of our patients with TOPORS-related RP. A preliminary review of the research gathered from this study was presented at the 2023 ARVO Annual Meeting in New Orleans, LA.

## 2. Methods

### 2.1. Patient Population

We performed a retrospective review of 416 patients who were diagnosed with inherited retinal disease at the WVU Eye Institute from January 2015 to June 2023. Most patients underwent comprehensive ophthalmic examination, including best-corrected visual acuity (BCVA), slit-lamp biomicroscopy, fundus photography, optical coherence tomography (OCT), visual field testing, and electrophysiology testing. The diagnosis of TOPORS related retinitis pigmentosa was based on clinical findings and confirmatory genetic testing. The study was approved by the Institutional Review Board of West Virginia University.

### 2.2. Genetic Analysis

Confirmatory genetic testing was completed at three different laboratories. Blueprint Genetics (MyRetinaTracker; Helsinki, Finland) with support from Foundation Fighting Blindness (Columbia, MD, USA), Invitae (ID Your IRD panel; San Francisco, CA, USA) with support from Spark Therapeutics (Philadelphia, PA, USA), and the Laboratory for Molecular Diagnosis of Inherited Eye Diseases (LMDIED Human Genetics Center—Houston, TX, USA) with support from The National Ophthalmic Disease Genotyping and Phenotyping Network eyeGENE NEI (Bethesda, MD, USA).

Samples processed by Blueprint Genetics were sequenced via high-quality custom capture technology and next-generation sequencing (NGS) for the IRB study protocol panel of 322 genes. Samples processed by Invitae were sequenced through similar technologies as part of a 248 to 330 gene panel. Confirmatory sequence analysis for samples processed by LMDIED were tested against a limited 9 gene panel for common mutations responsible for adRP after preliminary screening through eyeGENE at NEI. The members of Family 1 (F1) underwent testing with Blueprint Genetics. The members of Family 2 (F2) underwent testing with Invitae. Lastly, the members of Family 3 underwent testing with LMDIED. Within F3, Patient 7 (P7) underwent dual testing with LMDIED and Invitae.

## 3. Results

### 3.1. Identification of TOPORS Mutations

**Figure 1:**
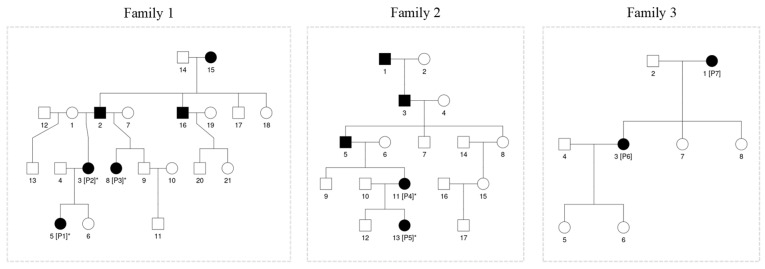
Shown above is the pedigree for the three study populations. All families demonstrate autosomal dominant inheritance patterns found in TOPORS related retinitis pigmentosa. The “*” represents probands with known mutational variants.

Among the 416 patients with inherited retinal disease at our institution, we identified a total of 7 patients from three separate families who had mutations in TOPORS (Figure 1). All identified patients were female and initially presented to our clinic with ages ranging from 12 to 70 years of age. A total of 7 out of 7 patients underwent confirmatory genetic testing. The average follow-up time in our patient population was (7.7 years). All mutations were heterozygous and segregated with the disease phenotype in each family. 

Of the study population, two families (F1 and F2) were found to have a previously reported frameshift mutation at c.2556_2557del (p.Glu852fs) that introduces a premature stop codon at position 871. One family (F3) had a novel nonsense mutation c.2431C>T (p.Gln811X) that replaces a glutamine codon into a stop codon at position 811.

### 3.2. Clinical Features of Patients with TOPORS Mutations

The exam findings and clinical characteristics of the 7 patients with TOPORS mutations are summarized in Table 1. Of note, missing data points are the result of incomplete documentation or loss to follow-up during retrospective chart review using the institutions electronic medical record. For symptomatic individuals, the average age of onset for vision loss was in their early 20′s. The most common complaint was night blindness, followed by progressive loss of peripheral vision and central vision in symptomatic individuals. The BCVA at the time of last follow up ranged from 20/20 to 20/60.

**Figure 2:**
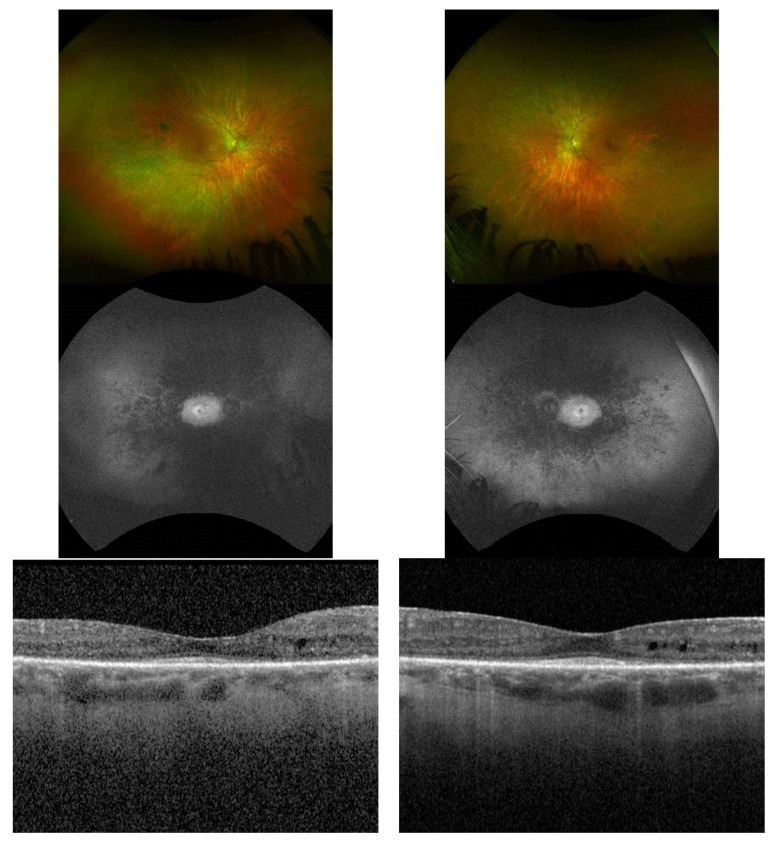
Ultra-Wide Field Fundus Images with FAF and OCT of the macula in P6 (Right Column—Right Eye, Left Column—Left Eye. The classic triad waxy optic nerve pallor, vascular attenuation, and bone spicules are all demonstrated in this image series. Notice the reduced peripheral fundus autofluorescence with a preserved central island in the macula. OCT findings of the macula demonstrate cystoid maculopathy with diffuse loss of the ellipsoid zone and outer retinal thinning.

**Figure 3:**
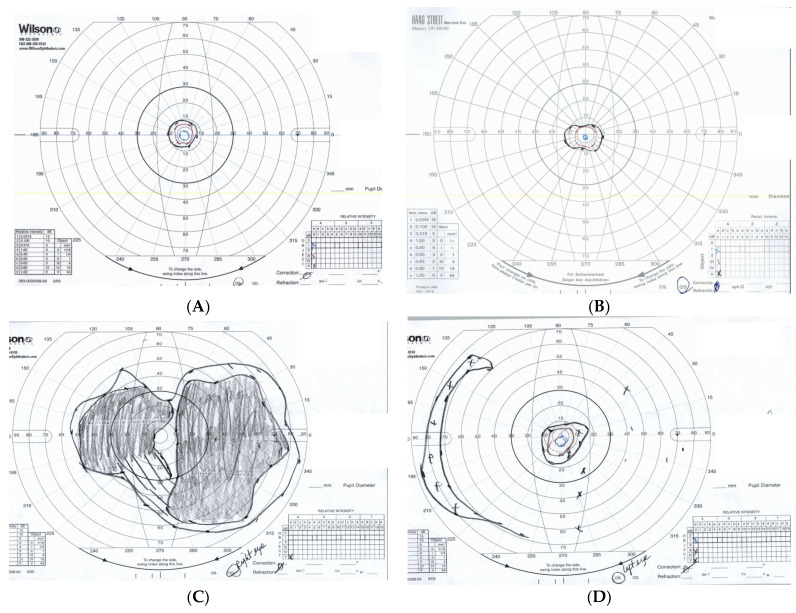
Goldmann Visual fields for P6 (above: (**A**)—Right Eye, (**B**)—Left Eye) and P3 (below: (**C**)—Right Eye, (**D**)—Left Eye). Notice the diffuse progressive loss in peripheral vision in both patients.

**Figure 4:**
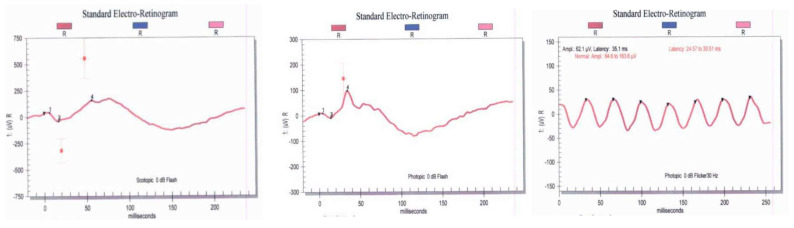

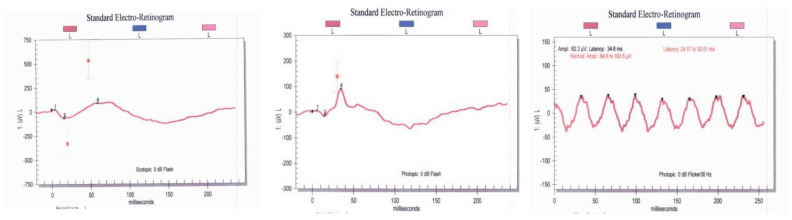
Full—field ERG demonstrating reduced a and b waves in a dark and light adapted setting ins subject P4. A wave amplitude is markedly attenuated which is consistent with the patients diminished photoreceptor function (Top—Right Eye, Bottom—Left Eye).

Fundus examination revealed typical RP features, such as bone spicule pigmentation, attenuated retinal vessels, optic disc pallor, and macular changes (Figure 2). There was significant thinning of the outer nuclear layer and disruption of the ellipsoid zone in all patients who underwent spectral domain OCT (SD-OCT) testing. CM was only present in two patients (P6 and P7). Patient P7 suffered from chronic bilateral vitreomacular traction (VMT) that eventually led to the development of a full thickness macular hole leading to rapid vision loss throughout the follow-up period. When available, all patients undergoing fundus autofluorescence photography (FAF) demonstrated diffuse reduced peripheral retina autofluorescence with a central fluorescent ring within the area of the macula correlating to remaining island of functional central photoreceptors at the fovea as seen in Figure 3. Visual field defects vary between individuals and the severity of peripheral vision loss correlated with age and the stage of the of RP. The visual field testing for P6 and P3 are shown below. In addition to abnormal FAF, ERG changes were documented in a subset of our patients who were able to undergo testing. Figure 4 demonstrates diminished full field ERG testing in P4 in primary in a dark-adapted scotopic environment consistent with the chief complaint of night blindness.

### 3.3. An Isolated Case of Full Thickness Macular Hole Leading to Rapid Vision Loss with Successful Repair

Within the subset of our patients with symptomatic RP secondary to TOPORS mutations, we report a 70-year-old female patient (P7) who developed a full thickness macular hole (FTMH) in her right eye measuring approximately 230 μm. The patient had a history of chronic vision loss in both eyes since the age of 30 years old. She also had a history of CM for years with concomitant VMT, which was refractory to topical carbonic anhydrase inhibitors and a series of intravitreal steroid injections. Genetic testing revealed that she carried the novel c.2431C>T (p.Gln811X) TOPORS mutation.

At the time of presentation for her full FTMH, her best corrected visual acuity (BCVA) was found to be 20/60 − 1 in the right eye and 20/50 − 1 in the left. She underwent 25-gauge pars plana vitrectomy (PPV) with epiretinal membrane (ERM) peel, internal limiting membrane (ILM) peel, and sulfur hexafluoride (SF6) gas tamponade with 7-day face down positioning in both eyes.

**Figure 5:**
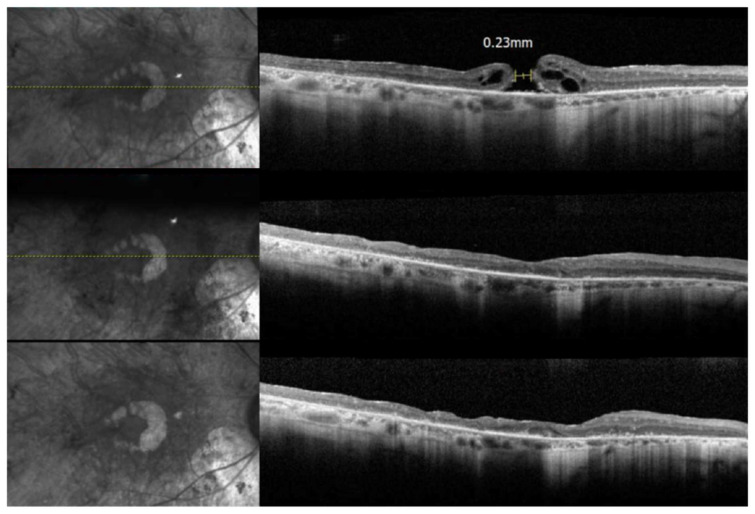
Spectral Domain OCT series of the right macula in P7. The first sequence (**top**) demonstrates the presence of a full thickness macular hole with adjacent cystoid macular edema and a faint epiretinal membrane. The second (**middle**) sequence was taken 1 month following the patient macular hole repair via pars plana vitrectomy with ERM and ILM peeling. In the third and final (**bottom**) sequence, we can appreciate persistent surgical closure of the full thickness macular hole 1 year following surgery. In all image series, there is diffuse outer retinal atrophy and photoreceptor loss consistent with late-stage retinitis pigmentosa.

In the first OCT sequence (Figure 5), the patient presented with a full thickness macular hole measuring 230 μm in diameter in the right eye. Her BCVA was 20/60 − 1 and her Goldmann visual field (GVF) showed less than the central 10° in the affected eye. In the third sequence, one year after the surgery, the macular hole was closed and the foveal contour was restored in the right eye. Her BCVA improved to 20/40 and she reported subjective improvement in central metamorphopsia which was also demonstrated on interval Amsler gird testing. The macular hole was found to remain closed at the time of her 3-year follow up.

## 4. Discussion

Mutations in TOPORS are a rare cause of autosomal dominant retinitis pigmentosa, with only a few families reported worldwide. In this study, we identified 7 patients from 3 families with TOPORS mutations in a cohort of 416 patients with inherited retinal disease from rural Appalachia treated at our institution. This represents the prevalence of TOPORs related RP to be 1.68% of adRP cases in our cohort similar to the estimated prevalence of 1% reported by Daiger, further emphasizing the rare nature of TOPORs related disease [1].

We identified a novel nonsense mutation c.2431C>T (p.Gln811X) in one family (F3), which is predicted to replace a glutamine codon with a stop codon, truncating the TOPORS protein by about 233 amino acids. Similar to previously described mutations, early truncation of the protein within exon 3 leads to deterioration of the Really Interesting New Gene (RING) finger motif function that is essential for ubiquitin ligase activity. Previous studies have shown that mutations in this domain impair the ubiquitination and degradation of p53, a tumor suppressor protein that regulates cell cycle and apoptosis [5]. It is possible that this mutation affects the stability and function of p53 in photoreceptors, leading to their degeneration. This mutation also agrees with the finding by Wang et al. that amino acid residues damaged at positions 807 to 867 which results in pathologic TOPORS phenotypes [8]. At the time of publication, only one other similar mutation reported by Invitae to NIH’s ClinVar database has been documented at c.2431C>G (p.Gln811Glu). This single nucleotide mutation has been described at the same location which replaces a glutamine with glutamic acid residue at the 811 position, which as of now is listed as a mutation of uncertain significance [9].

The two other families (F1, F2) had a previously reported frameshift mutation c.2556_2557del (p.Glu852fs), which is one of the most common TOPORS mutations found in adRP patients [1,7,10]. This nonsense mutation also results in a premature stop codon at position 871, which may lead to early degradation of the mRNA or a truncated protein with an absent RING finger domain.

The clinical features of our patients with TOPORS mutations appear to be consistent with previous reports of TOPORS-related RP. In most cases, disease onset was early, with night blindness being the first symptom. Similar to other aggressive RP, progression was severe, with the loss of peripheral then central vision leading to legal blindness by the fifth or sixth decade of life. Fundus appearance was also consistent with that of typical RP, such as bone spicule pigmentation, vessel attenuation, optic disc pallor, and pigmentary macular changes. On SD-OCT, findings revealed thinning of the outer nuclear layer and irregularity of the ellipsoid zone indicating photoreceptor loss. The visual field defects varied between individuals and the severity of peripheral vision loss correlated with age and the stage of the RP.

CM was present in two patients (P6 and P7). This appears to be a common complication of RP that can affect visual acuity and quality of life in patients with already limited vison. Interestingly enough, CM was not seen in the other families. Diabetes and other renal dysfunctions which are known independent causes of CM formation were not comorbid conditions in our study group. This may suggest that the patients in novel mutation group (F3) may be more predisposed to CM formation. However, further evidence to support this claim may be confounded by VMT or other variables not accounted for in our analysis. CME typically can be treated with topical or systemic anti-inflammatory agents, carbonic anhydrase inhibitors, or other intravitreal injections [11]. However, treatment response is variable depending on the severity of disease and often affected by other underlying comorbidities.

In our study group, one patient developed a full thickness macular hole (FTMH), which can be a rare occurrence in RP patients. FTMH is primarily a byproduct of abnormal vitreomacular forces [12]. It is characterized by a defect in the foveal neurosensory retina that extends through all retinal layers and causes detrimental central vision loss. The pathogenesis of FTMH in RP patients is not well understood, but has been previously described to be related to chronic VMT, ERM formation, or retinal atrophy [13,14]. FTMH can be surgically repaired by PPV with ILM peeling and supplemental gas tamponade. Repair of macular holes in RP patient is rare and long-term outcomes are poorly understood [13,14]. As in the case of our patient (P7), we performed a successful repair of a FTMH surgery, which resulted in improved visual acuity and persistent primary surgical success given persistent closure of the macular hole over three years of follow up.

The exact function of TOPORS in the retina is still unknown. Prior research has demonstrated TOPORS importance in maintaining photoreceptor homeostasis and survival through its supportive ubiquitin and SUMO1 ligase activities [5,6,15]. Mutations in TOPORS cause an aggressive form of RP compared to other adRP genes, which may reflect its involvement in multiple cellular pathways and processes as previously described. Further studies are needed to define the exact pathologic mechanisms underlying TOPORS-related RP and to identify potential therapeutic targets.

At the time of publication, the c.2431C>T (p.Gln811X) mutational variant demonstrated in F3 was recently changed in classification from a variant of uncertain significance (VUS) to “Likely Pathogenic” by Invitae in the latest report received in December 2023. Family 3 was affected by this variant had demonstrated early onset and aggressive RP in our study. Not all variants present in a gene cause disease, and some may be benign polymorphisms [2,9,15,16,17,18]. Similar to prior studies, premature translational stop signals in TOPORS related RP at exon 3 have shown to be pathologic, which provides additional evidence for the pathogenicity of our novel mutation [8].

## 5. Conclusions

In conclusion, we report the prevalence and characterization of TOPORS related RP mutations in a cohort of patients with inherited retinal disease from rural Appalachia. In our study population, we identified a novel nonsense TOPORS mutation c.2431C>T (p.Gln811X) in one family and a previously described frameshift mutation c.2556_2557del(p.Glu852fs) in two other unrelated families with pathologic adRP. Both of these mutations would appear to truncate the TOPORS protein and impair its E3 ubiquitin ligase activity, which may affect the stability and function of supportive cell cycle regulatory proteins in photoreceptors. We were also able to confirm that TOPORS-related RP is an early onset and severe form of RP that leads to legal blindness by the fifth or sixth decade of life in our unique study population.

The fundus appearance, OCT findings, ERG responses, and visual field defects were consistent with typical RP. CM was present in two patients and FTMH was present in one patient. We also describe a case of successful FTMH surgery in one of our patients with the novel mutational variant (P6), which resulted in improved visual acuity and persistent anatomical closure of the macular hole after three years of follow up. Our findings expand on the existing fund of knowledge related to the genetic and phenotypic spectrum of TOPORS-related RP, providing insights into its pathogenesis and management. Further studies are needed to elucidate the molecular mechanisms underlying TOPORS-related RP and to identify a potential for therapeutic targets or the possibility of gene therapy in the relative future.

## Figures and Tables

**Table 1:** A relative overview of demographics from the study population. Unavailable or incomplete information is listed with “NA”. ADHD = Attention-Defcit/Hyperactivity Disorder. GAD = Generalized Anxiety Disorder. IBS = Irritable Bowel Syndrome. GERD = Gastroesophageal Reflux Disease. PSC = Posterior Subcapsular Cataract. CME = Cystoid Macular Edema. PCIOL = Posterior Chamber Intraocular Lens. ERM = Epiretinal Membrane. FTMH = Full-Thickness Macular Hole. PTMH = Partial Thickness Macular Hole. GVF = Goldmann Visual Field.

ID	Family	Sex	Age at Last Follow Up	Age of Onset	Follow Up Time	Past Medical History	Past Ocular History	TOPORS Mutational Variant	BCVA OD	BCVA OS	Visual Field Testing
P1	F1	F	12	NA	2 Years	Autism, ADHD, Gait abnormality	None	c.2556_2557del	20/30 − 1	20/25 + 2	Mild Restriction OD. Smallest target seen is I3e.
P2	F1	F	35	20 s	2 Years	GAD, Smoker	Dry Eye	c.2556_2557del	20/25 − 1	20/40	NA
P3	F1	F	32	18	13 Years	IBS, GAD, Endometriosis	None	c.2556_2557del	20/25 − 1	20/25 − 1	About 80 central vision OU. Smallest target seen is V4e OD and I4e OS.
P4	F2	F	43	20 s	4 Years	GERD, Hypothyroidism	PSC OU	c.2556_2557del	20/25 − 1	20/25 − 1	Ring scotoma OU. Smallest target seen is I4e.
P5	F2	F	25	20 s	2 Months	GAD	Photophobia	c.2556_2557del	20/20	20/20	NA
P6	F3	F	51	20s	17 Years	Nephrolithiasis, Smoker, GAD	PSC OD, CME OU, PCIOL OD	c.2431C>T	20/60 − 1	20/60 + 2	GVF OU limited to central 10 degrees. Smallest target is I4e.
P7	F3	F	70	20 s	16 Years	Nephrolithiasis, Hypertension, Hyperlipidemia	PCIOL OU, Hx of PSC OU, CME OU, ERM OU, FTMH OD, PTMH OS	c.2431C>T	20/60	20/40	GVF of less than 10 degrees OU. Smallest target seen is V4e.

## Data Availability

Not applicable.
